# Why There Is No Poor Law

**Published:** 1920-07-10

**Authors:** 


### Hospital Problems in India.

### Why There is no Poor Law.

Calcutta Municipal Council has been asked by the Bengal Government to consider the question of a refuge for incurables. In this connection the council's health officer submitted a statement declaring that the proposal to establish a refuge for incurables put forward by the Surgeon-General with the Government of Bengal on the recommendation of Lt.-Col. Leventon, I.M.S., is one of those practical proposals which make one ask why it was not suggested long ago. Were it not for the joint family system, India would never have been able to exist so long without a Poor Law and its infirmaries and system of relief.

Whilst many aged and infirm are cared for by relatives, the figures given by Lt.-Col. Leventon show that a considerable number are in urgent need. As superintendent of a large hospital he is mainly concerned with incurables, and points out that these occupy beds which could be more useful in the treatment of cases where there is a prospect of relief. Such an institution as that proposed would have to provide for aged paupers no less than for cases discharged from the hospitals as incurable. It would have to be an almshouse as well as a refuge for incurables.

In view of recent suggestions that the Corporation should embark on an extensive programme of municipal hospitals and dispensaries involving a very heavy expenditure, says the health officer, the Corporation should carefully consider their general policy before committing themselves to any big schemes.

A scheme for the provision of one, if not two, large infectious hospitals outside the city is now under consideration. Whilst the Corporation will probably receive substantial aid from Government this project alone will involve a large sum of money. If the present proposal is also taken up—and it is, he thinks, obvious that it is the duty of the Corporation to provide accommodation for incurables and the aged poor—should not the Corporation limit its operations as regards hospitals to large institutions of this type which clearly come within their province?

The Municipal Council has agreed to consider the establishment of such a refuge along with its proposal for establishing a home for beggars in order to mitigate the beggar nuisance in the city.

